# Individualized medicine using 3D printing technology in gynecology: a scoping review

**DOI:** 10.1186/s41205-023-00169-9

**Published:** 2023-03-17

**Authors:** Carly M. Cooke, Teresa E. Flaxman, Lindsey Sikora, Olivier Miguel, Sukhbir S. Singh

**Affiliations:** 1 Department of Obstetrics, Gynecology and Newborn Care, University of Ottawa, Ottawa, Ontario Canada; 2 Department of Clinical Epidemiology, Ottawa Hospital Research Institute, Ottawa, Ontario Canada; 3 Department of Radiology, Radiation Oncology and Medical Physics, University of Ottawa, Ottawa, Ontario Canada; 4 Health Sciences Library, University of Ottawa, Ottawa, Ontario Canada; 5 Department of Obstetrics and Gynecology, The Ottawa Hospital, Riverside Campus, 1967 Riverside Dr., 7th Floor, Ottawa, Ontario K1H 7W9 Canada

**Keywords:** Gynecology, Patient specific, Scoping review, 3D printing

## Abstract

**Objective:**

Developments in 3-dimensional (3D) printing technology has made it possible to produce high quality, affordable 3D printed models for use in medicine. As a result, there is a growing assessment of this approach being published in the medical literature. The objective of this study was to outline the clinical applications of individualized 3D printing in gynecology through a scoping review.

**Data sources:**

Four medical databases (Medline, Embase, Cochrane CENTRAL, Scopus) and grey literature were searched for publications meeting eligibility criteria up to 31 May 2021.

**Study eligibility criteria:**

Publications were included if they were published in English, had a gynecologic context, and involved production of patient specific 3D printed product(s).

**Study appraisal and synthesis methods:**

Studies were manually screened and assessed for eligibility by two independent reviewers and data were extracted using pre-established criteria using Covidence software.

**Results:**

Overall, 32 studies (15 abstracts,17 full text articles) were included in the scoping review. Most studies were either case reports (12/32,38%) or case series (15/32,47%). Gynecologic sub-specialties in which the 3D printed models were intended for use included: gynecologic oncology (21/32,66%), benign gynecology (6/32,19%), pediatrics (2/32,6%), urogynecology (2/32,6%) and reproductive endocrinology and infertility (1/32,3%). Twenty studies (63%) printed 5 or less models, 6/32 studies (19%) printed greater than 5 (up to 50 models). Types of 3D models printed included: anatomical models (11/32,34%), medical devices, (2/32,6%) and template/guide/cylindrical applicators for brachytherapy (19/32,59%).

**Conclusions:**

Our scoping review has outlined novel clinical applications for individualized 3D printed models in gynecology. To date, they have mainly been used for production of patient specific 3D printed brachytherapy guides/applicators in patients with gynecologic cancer. However, individualized 3D printing shows great promise for utility in surgical planning, surgical education, and production of patient specific devices, across gynecologic subspecialties. Evidence supporting the clinical value of individualized 3D printing in gynecology is limited by studies with small sample size and non-standardized reporting, which should be the focus of future studies.

**Supplementary Information:**

The online version contains supplementary material available at 10.1186/s41205-023-00169-9.

## Introduction

Recent advancements in three-dimensional (3D) printing technology have facilitated the production of 3D printed models of exemplary quality. Continued reductions in operating costs and time to generate 3D printed models has increased feasibility and gained considerable interest from the medical field. 3D printed models can be scaled to size, and display fine details, closely resembling human anatomy. As a result, there is an increasing body of literature reporting on the clinical applications of 3D printing in medicine.

In high-fidelity 3D printing protocols, segmentation software is used to convert high quality 2-dimensional (2D) Magnetic Resonance (MR), Computed Tomography (CT) or ultrasound (US) images to 3D digital models, which can then be printed [1]. Hence, 3D printed models have the ability to be patient specific, with clinical applications in personalized medicine. In gynecology, 3D printed models can depict patient-specific female pelvic anatomy and gynecologic pathology, which may benefit physicians, trainees, and patients in their understanding of complex disease and management options.

With a growing body of literature in the area of 3D printing, there has been a need to summarize the data on 3D printing and develop clinical recommendations for its use. Systematic reviews have outlined the applications of 3D printing in surgery, identifying advantages including, better visualization of anatomy for pre-operative planning, improved operative outcomes, and decreased surgical time [2, 3]. As well, there have been studies which have reviewed the uses of 3D devices within specific surgical specialties such as orthopedics, spinal surgery, neurosurgery, plastics, and urology [4–8]. However, challenges in summarizing the data has been reported [9] such that the overall efficacy and effectiveness of 3D printed models across medical specialties remains unknown due to the breadth of uses, lack of comparable hypotheses, and non standardized reporting of outcomes across the literature [9].

## Objectives

A broad range of clinically meaningful applications for 3D printing in gynecology have been identified in the literature. The primary objective of this study is to systematically report the clinical applications of individualized 3D printing in gynecology. Additional objectives will be to summarize the production process for printing patient specific 3D printed models and determine the feasibility of personalized 3D printing in gynecology. We have chosen to use a scoping review to summarize our data, considering the challenges with performing systematic reviews on the topic of 3D printing in medicine [9] and mainly related to the heterogeneity of relevant studies.

## Methods

### Eligibility criteria, information sources, search strategy

A systematic review of the published literature was conducted to evaluate the uses of 3D printing in gynecology. Inclusion criteria consisted of publications up to and including 31 May 2021, of all study designs, which were published in English, had a gynecologic context and involved production of patient specific 3D printed models. Publications involving 3D imaging alone, without patient-specific 3D model production; where 3D printing was used for bioprinting, scaffolding, tissue engineering; or where 3D printing was used in a purely obstetrical context (i.e for fetal imaging, investigating fetal pathology), were excluded.

Four medical databases (Medline, Embase, CENTRAL, Scopus) and grey literature were searched using search terms which included “3D printing,” “gynecology” and relevant anatomic structures (vagina, cervix, uterus, fallopian tubes, ovaries, pelvic floor, ureters, urethra) or derivatives of these terms (Supplementary Material).

### Study selection

Studies were manually screened and assessed for eligibility by two independent reviewers, (CC, TF) initially by title and abstract review and subsequently by full text review.

### Data extraction

All data from studies selected for inclusion was extracted using a pre-established data extraction form. Disagreements between reviewers regarding study screening, eligibility, and data extraction were settled through discussion and consensus between the reviewers. Screening and data extraction was performed using the online platform Covidence. The study followed PRISMA protocol for scoping reviews [10].

### Assessment of risk of Bias

NA

### Data synthesis

The primary outcome was clinical applications of individualized 3D printing in gynecology. Additional outcomes assessed were 1) the production process used for producing 3D printed models (software, 3D printer, printing materials), 2) measures of feasibility (3D printing costs, production time). A descriptive approach for data synthesis was used.

## Results

### Study selection

**Fig. 1:**
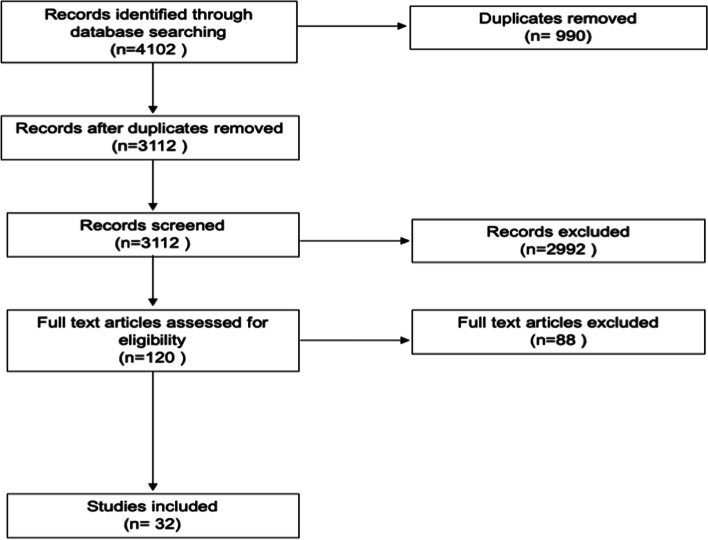
PRISMA flowchart

Our search yielded 4102 studies, of which 990 duplicates were removed, leaving 3112 studies to be screened. Title and abstract screening was performed by the reviewers leaving 120 studies for assessment of full text for eligibility. Eighty-eight studies were excluded for the following reasons: models were not patient specific (52), articles were duplicates (17), not the correct patient population (8), models were not printed (7), not in English (2), non-human models (1), and/or could not be accessed (1). In total 32 studies were included for review. PRIMSA flowchart can be seen in Fig. 1.

### Study characteristics

Of the 32 studies reviewed, 13 (41%) were case series, 12 (38%) were case reports, 4 (13%) were cohort studies, 2 (6%) were controlled trials (1 randomized and 1 non randomized) and 1 (3%) was a retrospective study. Nineteen studies (59%) were full text articles and the remaining 13 (41%) were conference abstracts. Studies were carried out in 13 different countries, with the most common places being China (9), The United States (6) and Canda (4). Studies were performed from 2014 to most recent. Most studies (21, 66%) printed 5 or less models. Seven studies (22%) printed greater than 5, (up to 50 models) and 4 (13%) studies did not specify the number of models produced. Additional study characteristics can be seen in Tables 1 and 2.

**Table 1:** Study characteristics

First author, date	Publication Type	Study Design	Sub-specialty	Patient Population	3D Printed Product	Intended Use	Intended User	Key Study Findings*
Ajao, 2017 [11]	Full text	Case report	BEN	Endometriosis	Anatomical model	Anatomical comprehension	MD	The 3D model accurately demonstrated the relationship of the endometriotic nodule to the patient’s anatomy
Baek, 2016 [12]	Full text	Case report	ONC	Cervical cancer	Anatomical model	Anatomical comprehension; surgical planning; patient education	MD; PT	3D models facilitate patient education and assist surgeons to plan operative intervention
Barbosa, 2019 [13]	Full text	Case series	REI	Infertility	Anatomical model	Surgical planning; improving assisted reproductive techniques	MD	3D models are feasible and can improve assisted reproductive techniques, assist in surgical planning
Barsky, 2018 [14]	Full text	Case report	URO	Stress urinary incontinence	Other medical device	Customized pessary for stress urinary incontinence	PT	A customized 3D printed pessary for treatment of stress urinary incontinence was successfully produced and inserted
Chang, 2018 [15]	Conference abstract	Case series	ONC	Locally advanced cervical cancer	Brachytherapy device	Brachytherapy treatment	MD	3D printing technology enabled precise apposition of applicators and dosimetry for image guided cervical cancer brachytherapy
Chen, 2017 [16]	Conference abstract	Case series	BEN	Placenta percreta	Anatomical model	Anatomical comprehension; surgical planning	MD; PT	The 3D printed model facilitated accurate assessment of placenta percreta and surgical planning, to improve maternal and fetal outcomes
Flaxman, 2020 [17]	Conference abstract	Case series	BEN	Uterine fibroids	Anatomical model	Anatomical comprehension; surgical planning	PT	3D printed models increased surgeon’s understanding of complex anatomy and impacted their surgical plan
Hadden, 2018 [18]	Conference abstract	Case series	PED	Congenital Mullerian anomalies	Anatomical model	Anatomical comprehension; provider education	PT; TR	3D models increased gynaecologists’ understanding of congenital Mullerian anomalies and surgical confidence
Jiang, 2020 [4]	Full text	Cohort study	ONC	Central pelvic recurrent gynecologic cancer	Brachytherapy device	Brachytherapy planning and treatment	MD	3D printed individual template based high dose rate interstitial brachytherapy is feasible and efficient, permitting delivery of localized interstitial brachytherapy
Kudla, 2019 [19]	Conference abstract	Case report	ONC	Locally recurrent endometrial cancer	Brachytherapy device	Brachytherapy treatment	MD	The custom applicator improved the quality and ease of delivery of interstitial vaginal brachytherapy
Laan, 2019 [20]	Full text	Case series	ONC	Gynaecologic cancer	Brachytherapy device	Brachytherapy treatment	MD	A personalised vaginal topography-based 3D printed for brachytherapy needle applicators, derived from patient MRI data was successfully designed and produced
Lindegaard, 2016 [21]	Full text	Case report	ONC	Locally advanced cervical cancer	Brachytherapy device	Brachytherapy treatment	MD	3D printing enabled a high degree of individualisation and exemplified superior dose distribution in brachytherapy treatment of stage IVA cervical cancer
Logar, 2019 [22]	Full text	Cohort study	ONC	Locally advanced primary/recurrent gynecologic cancer	Brachytherapy device	Brachytherapy treatment	MD	With use of 3D printed applicators, all dose volume parameters for clinical target volume improved without compromising dose constraints for organs at risk
Logar, 2020 [23]	Conference abstract	Case series	ONC	Vaginal/recurrent endometrial cancer	Brachytherapy device	Brachytherapy treatment	MD	An individually-designed multi-channel vaginal applicator was well tolerated, increased target coverage in advanced tumours, minimized trauma to surrounding tissue
Mackey, 2019 [24]	Full text	Case report	BEN	Uterine fibroids	Anatomical model	Anatomical comprehension; surgical planning	MD	The 3D-printed model facilitated cesarian section planning and use was associated with good maternal/fetal outcomes.
Mohammadi, 2021 [25]	Full text	Case report	ONC	Locally advanced cervical cancer	Brachytherapy device	Brachytherapy treatment	MD	High-temp resin with SLA 3D patient-specific multi-channel cylindrical applicators show mechanical accuracy and effective dosimetry
Pavan, 2021 [26]	Full text	Case report	URO	Mullerian agenesis; MRKH syndrome	Other medical device	Post-surgical customized vaginal mold	PT	Functional, histological and anatomical results were reached with the 3D printed tailored mold
Petric, 2019 [27]	Conference abstract	Cohort study	ONC	Locally advanced cervical cancer	Brachytherapy device	Brachytherapy treatment	MD	The use of 3D printed tandem needle template for image guided brachytherapy in locally advanced cervical cancer allowed successful management of disease
Qu, 2017 [28]	Conference	Case report	ONC	Recurrent cervical cancer	Brachytherapy device	Brachytherapy treatment	MD	The 3D printed individual applicator facilitated precise planning and decreased complications
Qu, 2019 [29]	Conference abstract	Non-randomised control trial	ONC	Pelvic wall recurrent gynecologic cancer	Brachytherapy device	Brachytherapy treatment	MD	When used for preoperative planning, both 3D-printed non coplanar template (3D-PNCT) and 3D-printed coplanar template achieve prescription dose, 3D-PNCT was more safe
Qu, 2021 [30]	Full text	Retrospective study	ONC	Non-central pelvic recurrent gynecologic cancer	Brachytherapy device	Brachytherapy treatment	MD	3D-printed non coplanar template assisted CT-guided 125I-seed ablative brachytherapy is a safe and feasible treatment
Reddy, 2019 [31]	Conference abstract	Case series	BEN	Uterine fibroids	Anatomical model	Anatomical comprehension; surgical planning	MD	Patient-specific 3D models facilitated preoperative and intraoperative planning for laparoscopic myomectomy
Sayed Aluwee, 2017 [32]	Full text	Case series	ONC	Endometrial cancer	Anatomical model	Anatomical comprehension; surgical planning; patient education	MD; PT	Personalized uterine 3D physical models using 3D printing and mold casting methods based on 3D MR images are useful for planning by surgeons, and patient communication
Sekii, 2018 [33]	Full text	Case series	ONC	Recurrent cervical cancer	Brachytherapy device	Brachytherapy treatment	MD	3D printing templates designed inversely have potential to assist in interstitial brachytherapy for vaginal tumors
Semeniuk, 2021 [34]	Full text	Case series	ONC	Gynecologic cancer	Brachytherapy device	Brachytherapy treatment	MD	Patient-specific cylinders provide comparable dose to the target, with advanced healthy tissue sparing
Sethi, 2014 [35]	Conference abstract	Case report	ONC	Endometrial cancer	Brachytherapy device	Brachytherapy treatment	MD	Successful production of a patient specific vaginal cylinder applicator for high-dose-rate intracavitary brachytherapy
Sethi, 2016 [36]	Full text	Case series	ONC	Primary/recurrent endometrial cancer	Brachytherapy device	Brachytherapy treatment	MD	Successful production of biocompatible, sterilizable, custom applicators for gynecologic brachytherapy
Wadi-Ramahi, 2018 [37]	Conference abstract	Case report	ONC	Gynaecologic cancer	Brachytherapy device	Brachytherapy treatment	MD	Patient-specific 3D printed molds facilitated personalized brachytherapy, specific to patient and tumor anatomy
Wang, 2020 [38]	Full text	Cohort study	ONC	Cervical cancer	Anatomical model	Anatomical comprehension; patient education	MD; PT	3D printed models can display patient anatomy and pathology to guide individualized brachytherapy for cervical cancer and communicate with patients
Yuan, 2019 [39]	Full text	Randomized control trial	ONC	Recurrent cervical cancer	Brachytherapy device	Brachytherapy treatment	MD	The 3D printed minimally invasive guidance template-assisted treatment provided dosimetry advantage
Zhao, 2019 [40]	Conference abstract	Case series	ONC	Retroperitoneal lymph node metastasis in gynecologic cancer	Brachytherapy device	Brachytherapy treatment	MD	3D printing template in seed implantation provides accurate positioning for treating retroperitoneal lymph node metastasis in gynecological oncology
Zhao, 2019 [41]	Full text	Case report	ONC	Cervical cancer	Brachytherapy device	Brachytherapy treatment	MD	A higher dose coverage of the target and better sparing of the organs at risk can be achieved by using a3D-printed, individualized cylinder

Sub-specialty: *BEN* benign gynecology, *ONC* gynecologic oncology, *REI* reproductive endocrinology and infertility, *URO *Urogynecology, *PED* Pediatric Gynecology. Intended user: *MD* physicians, *PT* patients, *TR* trainees

*Key findings were paraphrased directly from the study manuscript

**Table 2:** 3D-printed model production specifics

First author, date	Data Source	Software	3D Printer	3D Printing Material	Cost per Model (USD)	Production Time	No. of Models
Ajao, 2017 [11]	MRI	Mediprint	PolyJet J750, Stratasys	NS	NS	NS	1
Baek, 2016 [12]	CT	NS	Objet 260 CONNEX 3D printer, Stratasys	NS	NS	NS	1
Barbosa, 2019 [13]	MRI	3D PolyJet Studio; GrabcaD Print	Polyjet J750, Stratasys	Veroclear rGD810; Vero Magenta rGD 851; Tango Plus FlX930	NS	86mins – 30 hrs	4
Barsky, 2018 [14]	Trial/error	SolidWorks	Fused deposition modeling printer (model NS)	Polylactic acid	$10.94	2 hrs	3
Chang, 2018 [15]	CT/MRI	NS	Fortus 450mc, Stratasys	Polymer materials	NS	NS	5
Chen, 2017 [16]	MRI	NS	NS	NS	NS	NS	2
Flaxman, 2020 [17]	MRI	NS	NS	Resin	NS	NS	5
Hadden, 2018 [18]	MRI	NS	NS	NS	NS	NS	NS
Jiang, 2020 [4]	CT/MRI	Materialise Mimics, Geomagic	LITE450HD-B, Shanghai Liantai Technology Co Ltd.	Medical curing resin	NS	NS	32
Kudla, 2019 [19]	MRI	Eclipse; SolidWorks; Brachyvision	NS	NS	NS	NS	1
Laan, 2019 [20]	MRI	Oncentra, 3D Slicer; SolidWorks; MeVi-sLab; MatLab	Digital light processing (DLP)-based printer (Perfactory 4 mini XL, Envisiontec)	Liquid photopolymer resin	NS	NS	2
Lindegaard, 2016 [21]	CT/MRI	BrachyVision; Matlab; SolidWorks	Projet 3510 SD, 3D Systems	Visijet M3 Crystal, 3D Systems	NS	3 days (Print: 9 hrs)	2
Logar, 2019 [22]	MRI	BrachyVision	Formiga P100 3D printer	Biocompatible polyamide PA 2200	NS	NS	9
Logar, 2020 [23]	MRI	NS	Selective laser sintering technology (model NS)	Biocompatible polyamide PA	NS	NS	2
Mackey, 2019 [24]	MRI	3D Slicer	Ultimaker 3 Extended 3D printer	Polylactic acid filament	$35.00	49.5 hrs (printing)	1
Mohammadi, 2021 [25]	CT	Fusion 360; Meshmixer	UnionTech RS Pro 600	High-temp resinFLHTAM02 model, Formlabs Inc.	NS	4–5 hrs	NS
Pavan, 2021 [26]	Trial/error	NS	NS	Polylactic acid	NS	NS	NS
Petric, 2019 [27]	MRI	NS	NS	Biocompatible autoclavable material	NS	NS	13
Qu, 2017 [28]	CT	NS	NS	NS	NS	NS	1
Qu, 2019 [29]	NS	NS	NS	NS	NS	NS	NS
Qu, 2021 [30]	CT	Magics, Materialise	RS6000, Shanghai Liantaiv 3D Technology Company Inc.	NS	NS	NS	38
Reddy, 2019 [31]	MRI	Mediprint	PolyJet J750, Stratasys	Polymer	NS	NS	3
Sayed Aluwee, 2017 [32]	MRI	NS	Fused deposition modeling printer (model NS)	Polylactic acid with biodegradable thermoplastic	NS	3–5 days	5
Sekii, 2018 [33]	CT/MRI	CAD Software, Fusion 360 v.2.03174, Autodesk Inc.	Outsourced, DMM.com	Polycarbonate/acrylonitrile-butadiene-styrene (PC-ABS) polymer alloy	NS	Design: 2-3 hrs Print: 6-7 days	2
Semeniuk, 2021 [34]	CT	Eclipse; Oncentra; Matlab	NS	Biocompatible polymethyl methacrylate; tungsten-polylactic acid composite	NS	Design: 3 hrs Print: 3 hrs	2
Sethi, 2014 [35]	Physical exam	CAD software, Autodesk Inc.	Fortus 400mc, Stratasys	PC-ISO biocompatible thermoplastic	NS	NS	1
Sethi, 2016 [36]	Physical exam	CAD software, Autodesk Inc.	Fortus 400mc, Stratasys	PC-ISO thermoplastic	NS	NS	3
Wadi-Ramahi, 2018 [37]	CT	CAD software	NS	NS	NS	NS	2
Wang, 2020 [38]	MRI	3DDOCTOR	NS	Thermoplastics	NS	NS	50
Yuan, 2019 [39]	CT/MRI	Prowess Panther, Unicorn 3D template system	EP-A650	NS	NS	NS	11
Zhao, 2019 [40]	NS	NS	NS	NS	NS	NS	18
Zhao, 2019 [40]	CT	3ds Max, Autodesk Inc.; MakerBot	Replicator+, MakerBot	Polylactic acid	NS	NS	1

*MRI* magnetic resonance imaging, *CT* computed tomography, *NS* not specified

### Risk of bias

NA

### Synthesis of results

#### Primary outcome

##### Clinical applications and impact of personalized 3D printed models

3D printed models were intended for use by physicians (25/32, 78%), both physicians and patients (4/32, 13%), both physicians and trainees (1/32, 3%) or patients (2/32, 6%). Models were used in studies for each of the gynecologic subspecialties including, gynecologic oncology (23/32, 72%), benign gynecology (5/32, 16%), urogynecology (2/32, 6%), pediatric gynecology (1/32, 3%), and reproductive endocrinology and infertility (1/32, 3%). Patient pathologies studied included gynecologic cancer (23/32, 72%), uterine fibroids (3/32, 9%), Mullerian anomalies (2/32, 6%), endometriosis (1/32, 3%), placenta percreta (1/32, 3%), stress urinary incontinence (1/32, 3%), and infertility (1/32, 3%). In 20 (63%) studies, the patient specific 3D printed models being produced were brachytherapy templates/cylindrical applicators; in 10 (31%) studies they were anatomical models; and in 2 (6%) studies they were other medical devices. Specific 3D printed models produced in each study can be seen in Table 1.

#### Secondary outcomes

##### 3D printed model production and feasibility

Data sources used for production of the 3D printed models included: MRI (14/32, 44%), CT (7/32, 22%), both MRI and CT (5, 16%), physical exam (2/32, 6%), trial and error (2/32, 6%) or did not specify (2/32, 6%). Data software, 3D printers and 3D printing materials used varied across studies. The most commonly used 1) data software were Computer Aided Design (CAD) Software (4/32, 13%) and Solidworks (5/32, 16%); 2) 3D printers were Stratasys Fortus (3/32, 9%) and PolyJet J750 (3/32, 9%); and 3D printing material was polylactic acid (5/32, 16%). A large number of studies did not specify data software (12/32, 38%), 3D printer (13/32, 41%), or 3D printing materials (11/32, 34%) used. One study produced a 3D printed mold, from which multiple models could be produced.

3D printing costs were only provided by 2 (6%) studies and production time by 7 (22%) studies. Costs listed per model were $10.94 and $35 USD. Production time varied from 86 minutes to 5 days.

## Discussion

With a growing body of literature in the area of 3D printing and continuous advancements in its technology, there has been a need to summarize the data on 3D printing and its clinical applications in medicine. We performed a scoping review to systematically report on the clinical applications of individualized 3D printing in gynecology. Although a review on the role of 3D printing in gynecology has previously been published [13], this study was limited in its reporting of applications for reproductive surgery only. Furthermore, its search was limited to a single platform (Pubmed), yeidling only 11 studies, and lacked information on the feasibility and impact of 3D printing on patient outcomes in gynecology. Here, we present on themes regarding clinical applications of patient specific 3D printing in gynecology, as summarized below.

### Medical devices

Brachytherapy is an integral component of the management of both primary and recurrent gynecologic cancers. It facilitates the delivery of a high dose of localizaed radiation to a small volume tumor, while minimizing radiation dose to surrounding normal tissue [42]. To optimize treatment, selection of the most appropriate brachytherapy technique, intracavitary versus interstitial, and applicator, should be individualized based on the depth of invasion, distribution of disease, and patient specific anatomy [43]. A variety of applicator designs and sizes have been developed to limit patient discomfort while enhancing radiation dose distribution [42, 43]. However, still it remains a challenge to find an optimally fitting brachytherapy applicator for each patient’s individual anatomy and pathology [43].

Our scoping review has highlighted that patient specific 3D printed brachytherapy devices have been the most commonly studied individualized 3D printed model in gynecology in the literature to date [19–23, 25, 27, 29, 30, 33–37, 39–41, 44]. The 3D printed models produced and studied were mainly personalized vaginal brachytherapy cylinder applicators and or interstitial brachytherapy needle templates in a population of patients with gynecologic malignanies including primary vaginal cancer, locally advanced or recurrent cervical or endometrial cancer [19, 20, 22, 23, 25, 29, 30, 33–37, 39–41, 44]. In addition, some studies created 3D printed devices that could be personalized and used in combination with standardized applicators or templates [21, 27].

Some of the larger cohort studies provided clinically relevant results supporting the utility of individualized 3D printed devices for use in brachytherapy treatment of gynecological malignancies. Specifically, Logar et al. (2019) and Yuan et al. (2019) report increased radiation doses to the target volume and decreased dose to organs at risk, in patients with gynecologic malignancies previously treated with external beam radiation, when 3D printed individualized 1) vaginal applicators and 2) guidance templates, respectively, were used for brachytherapy treatment, in comparison to standardized devices [22, 39]. Similarly, 3D printed individualized brachytherapy trans-vaginal template/applicator +/− transperineal template facilitated high dose parameters, a high response rate (84.4% 1 month after completion), with no severe complications, in of a group of patients with central recurrent gynecologic malignancy in the study by Jiang et al. (2020) [44]. Further, Qu et al. (2021) showed that 3D-printed non-coplanar template (3D-PNCT)-assisted computed tomography (CT)-guided iodine-125 seed ablative brachytherapy could reduce the misalignment error and improve accuracy of needle puncture for non-central pelvic lesions [30].

These studies each used uniquely designed patient specific 3D printed brachytherapy applicators/templates for specific gynecologic oncology patient populations, and altogether suggest significant benefit to their use. Studies which can reproduce these results, and provide long term data on outcomes, while also investigating feasibility may facilitate wider spread use of these devices in a clinical setting in the future.

While the literature regarding the use of patient specific 3D printed personalized devices has been well explored in the context of brachytherapy applicators, there may be further utility of 3D printed personlized medical devices for other purposes. Barsky et al. (2018) showed that a patient specific silicone pessary produced from a 3D printed mold was effective in management of stress urinary incontinence and showed no short term complications [14]. Authors from another study, which was however excluded from this review due to it’s obstetrical context, similarly used 3D printing to produce a patient specific cervical cerclage pessary [45]. Unique utility was additionally shown by Pavan et al. where an individualized 3D printed vaginal mold was used by a patient with Mayer-Rokitansky-Küster-Hauser (MRKH) syndrome following McIndoe modified vaginoplasty, as a permanent dilator post-operatively promoting return to sexual function [26].

This scoping review has outlined excellent examples of patient specific medical devices in gynecology, including brachytherapy applicator/templates, pessaries, and a vaginal dilator. Other studies have presented approaches and assessed the feasability of using 3D printing to introduce multiple shapes and sizes of various gynecologic devices such that variations in patient anatomy can be better accomodated for. Examples include connector tubing for dilatation and evacuation [46], intrauterine balloons for management of post partum hemorrhage [47], vaginal speculums [48], and drug eluting intravaginal rings [49–54]. When applicable, create patient specific devices using 3D printing can have an even greater potential for best fit, which can improve their effectiveness and patient experience. Hence efforts should be made to continue to create, produce, and study personalized devices in gynecology further. Some challenges to the widespread production and use of patient specific devices are related to cost and time burden of production, and the requirement of approval from health regulatory bodies. But, larger studies showing effectiveness and safety may help to overcome some of these limitations.

### Surgical planning

Studies have also suggested a role for individualized 3D printed models for surgical planning. As initial proof of this concept, Ajao et al. and Mackey et al. produced high fidelity individualized 3D printed models which were shown to accurately represent gynecologic pathology (i.e endometriotic nodules or fibroids) in relation to the surrounding tissues, and closely correlated with patient anatomy at the time of surgery [11, 24].

Additional studies have outlined the the utility of patient-specific 3D printed models for surgical planning and intraoperative assistance further [12, 13, 16, 17, 31, 32] In preparation for benign gynecologic procedures, Flaxman et al. (2020) found that that the use of patient-specific 3D-printed models altered the surgeons’ perception of surgical difficulty, perceived risk for surgical complications, and planned hemostatic techniques, and increased their confidence in their pre-operative plan [17] and Chen et al. (2017) showed that the models decreased operative time and blood loss [16]. Baek et al. (2016) and Sayed Aluwee et al. (2017) reported that gynecologic oncologists had an increased comprehension of patient anatomy and pathology (eg. tumor size, shape, borders) [12, 32], and increased confidence in route of excision [12], with use of individualized 3D printed models, in preparation for oncologic surgeries. Finally, Barbosa et al. (2019) reported that patient specific 3D printed models provided novel information and assisted in planning of infertility procedures, including hyperoscopic myomectomy, septoplasty and embryo transfer, and assessment of ovarian reserve in preparation for IVF [13].

Overall, these studies highlight that in preparation for complex gynecologic procedures, across gynecologic subspecialties, personalized 3D printed models may provide additional infomation to the surgeon regarding patient specific anatomy and pathology, greatly assisting in the development of their surgical plan. While theoretically, with better preparation for the surgical procedure, it seems that there is the potential for the models to help to reduce complications and improve outcomes, none of the studies in this review were able to provide evidence to support this. Hence, studies are needed to further investigate surgical outcomes related to the use of patient specific 3D printed models for surgical planning to provide clearer evidence to the benefit of their use.

Two studies have also shown benefit of 3D printed patient specific models for brachytherapy planning [15, 38]. In these studies, 3D printed patient specific models were effective and non invasive for pre-planning brachytherapy in patients with cervical cancer [15, 38]. Physicians using the models, reported high fidelity and usefulness, and their overall evaluation of the cervical cancer model was 8.0 ± 0.8 points [38].

### Education

Personalized 3D printed models have also been investigated as an educational tool. In one study, patient specific 3D models of Mullerian anomalies were found to increase gynecologists’ understanding of Mullerian anomalies and their confidence in surgical correction [18]. There is also evidence that they may help to promote patient education [12, 32, 38]. Patients report greater understanding of their disease and radiotherapy treatment or surgical intervention with the assistance of the 3D printed models [12, 32, 38].

The literature regarding the utility of patient specific 3D printed models for educational purposes in this scoping review appears limited. However, during our review of the literature, we did note that there is more significant data regarding the use of non-patient specific 3D printed models in education in gynecology [55–57]. Unfortunately, these were excluded from our scoping review due to the non-patient specific nature of the 3D printed models. This has idenitified a need for a furture study to summarize the literature regarding 3D printing overall, inclusive of both patient specfic and non-patient specific models, for the purposes of trainee education in gynecology.

### Methodological considerations

Our study has identified a need for larger, higher quality studies and more consistent reporting on the topic of individualized 3D printing in gynecology. The majority of the studies in this scoping review were case reports or small case series which were proof of concept pilot studies. These studies have provided strong evidence that we now have the technology to produce patient specific 3D printed models in gynecology, and that there are many great uses possible. However, unfortunately the workflow process for production of the personalized 3D printed models including software, 3D printer, and materials used, as well as measures of feasibility, such as cost, and time for production were widely under-reported. As a result, reproducibility of these studies is limited. Further, the true feasibility of personalized 3D printed models remains unknown, as measures of feasibility were mainly unreported. Further, when they were reported, for example, cost per model of $10.94 and $35 USD, is misleading, as this does not account for the costs of the printer itself, and payment of the team who are needed to assist in preparing images for 3D printing. Further production time again was mainly unreported or else highly variable and non specific.

Finally, while the studies in this scoping review suggest clinical benefit to the use of patient specific 3D printed models, the data to support this was scant. Again, there was a focus on the ability to produce patient specific 3D printed models, but minimal data providing evidence to their impact on patient outcomes. In order for personalized 3D printing to be used in a widespread fashion in gynecology and supported by our heathcare system, we need studies which provide cost-to-benefit analysis and which provide evidence of their ability to improve patient outcomes. Hence, we are putting out a call for larger, experimental studies with clear and consistent reporting of feasibility measures on the topic of personalized 3D printing in gynecology, which will provide us with the data we need to promote their ongoing utility in this specialty.

## Conclusion

Overall, this study has highlighted that there are a number of studies on the topic of personalized 3D printing in gynecology currently available. Through our scoping review we have summarized the literature to date on the topic of personalized 3D printing in gynecology and outlined many novel and potentially practice changing uses across gynecologic subspecialties. Some of these uses have included personalized applicators/templates for brachtherapy in the management of gynecologic malignancies, and other customized medical devices, as well as patient specific models for surgical planning and patient and trainee education.

## Supplementary Information

**Additional file 1.** Search Strategy.

## Data Availability

Not available.
