# Supplementary material for: Individualized medicine using 3D printing technology in gynecology: a scoping review
Source: 3D Print Med. 2023 Mar 17;9:6. doi: 10.1186/s41205-023-00169-9 (PMC10024374; doi:10.1186/s41205-023-00169-9)
Supplement: Supplementary file 1 — Additional file 1. Search Strategy. [file 41205_2023_169_MOESM1_ESM.docx]

**APPENDIX 1**

**Medline**1. Gynecology/

2. "Obstetrics and Gynecology Department, Hospital"/

3. gynecolog*.tw,kw.

4. gynaecolog*.tw,kw.

5. obgyn.ti,ab.

6. fallopian tubes/ or ovary/ or uterus/ or vagina/ or vulva/ or ureter/ or urethra/ or urinary bladder/

7. Pelvic Floor/

8. Cervix Uteri/

9. (vagina* or cervix* or uterus* or vulva* or ureter* or urethra* or uterine* or ovary or ovaries or bladder*).tw,kw.

10. (fallopian adj1 tube*).tw,kw.

11. (pelvic adj1 (floor or floors)).tw,kw.

12. or/1-11

13. exp Printing, Three-Dimensional/

14. ((3D or 3-D or 3-dimensional or three dimensional) adj2 (print* or imaging* or image or images or model* or anatomy or rendering or digital)).tw,kw.

15. 13 or 14

16. 12 and 15

17. 16 not (Animals/ not (Animals/ and Humans/))

**Embase**

1. gynecology/

2. gynecolog*.tw,kw.

3. gynaecolog*.tw,kw.

4. obgyn.ti,ab.

5. pelvis floor/

6. exp vulva/

7. vagina/

8. female genital system/

9. fallopian tube/

10. ovary/

11. ureter/

12. urethra/

13. bladder/

14. uterus/

15. (vagina* or cervix* or uterus* or vulva* or ureter* or urethra* or uterine* or ovary or ovaries or bladder*).tw,kw.

16. (fallopian adj1 tube*).tw,kw.

17. (pelvic adj1 (floor or floors)).tw,kw.

18. or/1-17

19. exp three dimensional printing/

20. ((3D or 3-D or 3-dimensional or three dimensional) adj2 (print* or imaging* or image or images or model* or anatomy or rendering or digital)).tw,kw.

21. 19 or 20

22. 18 and 21

23. 22 not (Animals/ not (Animals/ and Humans/))

**CENTRAL**

1. Gynecology/

2. "Obstetrics and Gynecology Department, Hospital"/

3. gynecolog*.tw,kw.

4. gynaecolog*.tw,kw.

5. obgyn.ti,ab.

6. fallopian tubes/ or ovary/ or uterus/ or vagina/ or vulva/ or ureter/ or urethra/ or urinary bladder/

7. Pelvic Floor/

8. Cervix Uteri/

9. (vagina* or cervix* or uterus* or vulva* or ureter* or urethra* or uterine* or ovary or ovaries or bladder*).tw,kw.

10. (fallopian adj1 tube*).tw,kw.

11. (pelvic adj1 (floor or floors)).tw,kw.

12. or/1-11

13. ((3D or 3-D or 3-dimensional or three dimensional) adj2 (print* or imaging* or image or images or model* or anatomy or rendering or digital)).tw,kw.

14. 12 and 13
